# Enhanced Growth of Endothelial Precursor Cells on PCG-Matrix Facilitates Accelerated, Fibrosis-Free, Wound Healing: A Diabetic Mouse Model

**DOI:** 10.1371/journal.pone.0069960

**Published:** 2013-07-26

**Authors:** Meghana Kanitkar, Amit Jaiswal, Rucha Deshpande, Jayesh Bellare, Vaijayanti P. Kale

**Affiliations:** 1 National Centre for Cell Science, NCCS Complex, University of Pune Campus, Ganeshkhind, Pune, Maharashtra, India; 2 Department of Chemical Engineering, Indian Institute of Technology-Bombay, Powai, Mumbai, Maharashtra, India; Johns Hopkins University, United States of America

## Abstract

Diabetes mellitus (DM)-induced endothelial progenitor cell (EPC) dysfunction causes impaired wound healing, which can be rescued by delivery of large numbers of ‘normal’ EPCs onto such wounds. The principal challenges herein are (a) the high number of EPCs required and (b) their sustained delivery onto the wounds. Most of the currently available scaffolds either serve as passive devices for cellular delivery or allow adherence and proliferation, but not both. This clearly indicates that matrices possessing both attributes are ‘the need of the day’ for efficient healing of diabetic wounds. Therefore, we developed a system that not only allows selective enrichment and expansion of EPCs, but also efficiently delivers them onto the wounds. Murine bone marrow-derived mononuclear cells (MNCs) were seeded onto a PolyCaprolactone-Gelatin (PCG) nano-fiber matrix that offers a combined advantage of strength, biocompatibility wettability; and cultured them in EGM2 to allow EPC growth. The efficacy of the PCG matrix in supporting the EPC growth and delivery was assessed by various in vitro parameters. Its efficacy in diabetic wound healing was assessed by a topical application of the PCG-EPCs onto diabetic wounds. The PCG matrix promoted a high-level attachment of EPCs and enhanced their growth, colony formation, and proliferation without compromising their viability as compared to Poly L-lactic acid (PLLA) and Vitronectin (VN), the matrix and non-matrix controls respectively. The PCG-matrix also allowed a sustained chemotactic migration of EPCs in vitro. The matrix-effected sustained delivery of EPCs onto the diabetic wounds resulted in an enhanced fibrosis-free wound healing as compared to the controls. Our data, thus, highlight the novel therapeutic potential of PCG-EPCs as a combined ‘growth and delivery system’ to achieve an accelerated fibrosis-free healing of dermal lesions, including diabetic wounds.

## Introduction

Diabetes mellitus (DM) is a metabolic disorder characterized by mild to severe hyperglycemia, which adversely affects most organ systems. Ensuring a tight glycemic control is necessary to mitigate or retard these effects [1], as persistent high plasma glucose concentrations cause secondary complications such as diabetic nephropathy, retinopathy, neuropathy and impaired wound healing, among others [2]. Diabetes-induced impaired wound healing, alone, is the cause of majority of the extremity amputations worldwide, involving intense pre- and post-operative trauma, therefore, timely healing of diabetic wounds is imperative [3].

Active contribution of peripheral blood (PB)- or bone marrow (BM)-derived endothelial progenitor cells (EPCs) in the wound healing process via angiogenic/vasculogenic processes has been unequivocally documented in several studies [4]–[7]. Diabetes-induced hyperglycemia renders these EPCs dysfunctional and impairs their ability to effectively ‘home’ to the wound site. In this context, transplantation of non-diabetic EPCs isolated/cultured from bone marrow, cord blood as well as peripheral blood onto the diabetic wounds has led to successful and faster wound healing with an enhanced neo-vascularization [8], [9]. However, the prime challenge for implementation of this therapy, or of any cell therapy, lies in having a one-step culture-cum-delivery system for application at the wound site. Normally, EPCs from various sources are isolated and/or grown, characterized and then seeded onto biocompatible-scaffolds, which are then used as a system for delivering the EPCs onto diabetic wounds, either as a large single bolus or as multiple boli [10], to facilitate their participation in the healing process. Barring a few exceptions, most of these matrices neither support growth and/or proliferation of the attached EPCs, nor allow enrichment of EPCs from a heterogeneous seeding population like PBL−/BM-derived MNCs. Also, the majority of these matrices do not contribute to the regulation of EPC-delivery, a crucial factor in the efficient recovery of diabetic wounds.

Electro-spun matrices of Poly-Caprolactone (PC)-Gelatin (G), hereafter called as PCG, matrices have been used previously for attachment/growth of human endothelium, mesenchymal cells and nerve tissue with some degree of success. Some formulations of PCG matrices have also been used for the delivery of EPCs onto wound sites for enhanced wound coverage [11]–[20]. However, a formulation that supports selective growth of EPCs and their delivery onto diabetic wound sites has not been developed previously.

Although, PC alone is completely hydrophobic and hence does not allow cell attachment and proliferation [22], complexing it with various other compounds has resulted in biologically usable materials; one of them being gelatin (G). It is known that, gelatin, in a concentration-dependent manner, positively affects cellular adherence and proliferation. In the present study we fabricated an electro-spun matrix using PolyCaprolactone (PC) and Gelatin (G) at a novel ratio of 3∶1. The matrix fabricated at this ratio was found to provide an optimal strength for application purposes and gave a suitable degree of ‘wettability’ combined with biocompatibility. We further tested the potential of this PCG matrix as a substrate for adhesion, enrichment and growth of EPCs. We also evaluated its efficacy as a viable therapeutic option for diabetic wound healing in a standard murine diabetic model.

We found that the PCG matrix allowed cellular adhesion, proliferation as well as facilitated a sustained chemotactic migration of EPCs in vitro as well as in vivo. Most importantly, topical application of PCG-EPCs on diabetic wounds resulted in an accelerated and fibrosis-free wound healing.

We therefore propose this novel PCG-EPC system as a one-step “combined growth and delivery system” for direct application onto the dermal lesions, including the diabetic wounds. The dual advantage offered by this matrix in terms of its EPC growth-supporting property and its ‘easy to handle’ nature is of immense importance in clinics practising cell therapy in human subjects as well as in veterinary practice.

## Materials and Methods

### Test Systems

All animal procedures used complied with the international guidelines governing animal experimentation and were approved by the Institutional Animal Ethical Committee at NCCS, Pune (EAF/2011/B-170). Swiss/albino male mice (6 to 8 weeks old weighing at least 22 g), originally purchased from the Jackson Laboratory, USA and bred in an inbred colony at the National Centre for Cell Science, India, were used in the experiments. They were given free access to standard feed and water.

### Drugs, Chemical Reagents and Materials

Endothelial Growth Medium (EGM-2 SingleQuots) was from Lonza, Walkersville, MD, USA, while fetal bovine serum (FBS) and Dulbecco’s Modified Essential Medium (DMEM) were purchased from Invitrogen (Carlsbad, CA, USA). 3-(4, 5-Dimethyl-2-thiazolyl)-2,5-diphenyl-2H-tetrazolium bromide (MTT) and Vitronectin (VN) were from Sigma-Aldrich, (St Louis, MO, USA). All plasticware was from BD Falcon (Bukit Batok Crescent, Singapore).

### Fabrication of Matrix

Polycaprolactone (PC) (Sigma- Aldrich, St.Louis, MO, USA) with an average molecular weight of 80,000 Da and Gelatin (Type A) from porcine skin were used together with 1,1,1,3,3,3-hexafluoro-2-propanol (HFP) (Sigma- Aldrich, St. Louis, MO, USA). PC/Gelatin in a 3∶1 ratio (w/w) was dissolved in HFP to make a 12% final solution. This ratio has been optimized in our lab for maximal attachment and growth of cells. Increasing the proportion of polycaprolactone would decrease cellular attachment while increasing the proportion of gelatin would lead to a decrease in strength, compromising its applicability as a delivery substrate. This solution was stirred overnight at ambient temperature and was electrospun into nanofibers. For electro-spinning, the polymer solution was filled in a 5 ml plastic syringe (BD, India) connected to a blunt ended metallic needle (24 Gauge). The syringe was then loaded into a syringe pump (New Era Pump System Inc., USA) and electrospun at the rate of 1 ml/hour. The collector was covered with an aluminium foil. The needle tip to collector distance was maintained at 12.5 cm, and a voltage of 14 kV was applied. The entire process was conducted in a fume hood at ambient temperature of 27°C and 55% relative humidity. The electrospun scaffold was kept under vacuum overnight before performing further experiments. The matrix can be sterilized by simple methods such as gamma irradiation or 70% alcohol rinse. During the current experimental set-up, the matrix has been irradiated using gamma rays prior to use (8000 rads).

### Material Characterization of Scaffold using Scanning Electron Microscopy (SEM) and Transmission Electron Microscopy (TEM)

The morphology of the electrospun PCG nanofibers was examined by field emission scanning electron microscopy (JEOL, JSM-7600F) at an accelerating voltage of 15 kV. For SEM, the samples were cut into 5×5 mm squares, mounted on sample stubs, and coated with gold by sputter coating using an SC7640 Sputter Coater (Quorum Technologies Ltd, UK). The average fiber diameter of the scaffolds was determined from the SEM micrographs using image analysis software (ImageJ, National Institutes of Health, Bethesda, USA). For TEM, fibers of PCG were collected on TEM grids during the process of electro-spinning and further observed by transmission electron microscopy (TEM, Tecnai 20, Philips FEI, Netherlands) at a voltage of 100 kV.

For the fabrication of control poly-L-lactic acid (PLLA) electrospun matrix, PLLA (average molecular weight-300,000 Da, Polysciences, Inc. Warrington, USA) was dissolved in HFP to make an 8% (w/v) solution for electrospinning. The other process parameters were kept identical as for electro-spinning of PCG. PLLA matrix was also sterilised by gamma irradiation before use (8000 rads).

### Isolation, Culture and Characterization of EPCs

The EPC isolation protocol as described by Asahara et al., 1997, was followed with modifications. Briefly, mice were sacrificed by cervical dislocation and femurs were extracted aseptically [21]. These bones were flushed using plain DMEM, the cellular suspension was loaded onto HiSep LSM (HiMedia, Mumbai, India) and centrifuged for 30 min at 1500 rpm at ambient temperature. The buffy coat comprising of Mononuclear Cells (MNCs) was aspirated and washed twice with plain DMEM for 15 min at 1500 rpm, at ambient temperature. These cells were then counted and 1×10^6^ MNCs were seeded onto rat Vitronectin (VN) (Sigma Aldrich, MO, USA)-coated Petri-dishes and incubated for 48 h in DMEM supplemented with 10% FBS at 37°C with 5% CO_2_. After 48 h, the non-adherent fraction was collected and 1000 MNCs/well were seeded separately onto VN-coated wells (VN-EPCs: non matrix control), PLLA matrix (PLLA-EPCs: matrix controls) or PCG matrix (PCG-EPCs - test group) in 24 well plates or 35 mm dishes as per experimental requirement. Cells were cultured in EGM2 supplemented with 20% FBS and incubated for the next 14 d at 37°Cwith 5% CO_2_. The medium was replenished every 48 h. A patent has been filed for the said technique and the resultant final product (i.e. PCG- EPCs). (Filed patent number: 3343/MUM/2012.).

EPCs were identified by co-positivity of Ac-LDL uptake and *Ulex europeaus* Agglutinin 1 (UEA-1) binding. Briefly, adherent cells were washed thrice with 1×PBS (w/o Ca^+2^/Mg^+2^) and incubated with Alexaflour 488-labelled Ac-LDL (Invitrogen, Carlsbad, CA, USA) for 3 h at 37°C, washed with 1×PBS and thereafter fixed with chilled 2% buffered paraformaldehyde. These were then stained with 10 µg/ml TRITC- labelled *Ulex europaeus* Lectin (agglutinin 1) (Sigma Aldrich, St Louis, MO, USA). Dual positive cells were considered as EPCs and were counted in ten random non-overlapping fields by using an inverted fluorescence microscope and Image-pro software (Leica TCS SP5II, Leica Microsystems, Mannheim, Germany). To facilitate counting and improve reproducibility, all cellular nuclei were stained with DAPI.

### EPC Cell Adhesion Assay

EPCs (800 mMNCs/well – VN-coated, PLLA, PCG matrix sets as mentioned above) were cultured in EGM-2 supplemented with 20% FBS for 14 d at 37°C with 5% CO_2_ and then stained with crystal violet. Post-staining, the cells were thoroughly washed with 1×PBS and the dye was extracted using 2% SDS and optical density is measured at 550 nm. The resultant values were plotted against a standard curve and results were plotted in a graphical format. At least triplicate assays were performed for each sample. The adhered EPCs were also counted manually using a light inverted microscope (Olympus).

### EPC Colony Formation Assay

EPCs, (800 mMNCs/well – VN-coated, PLLA, PCG matrix sets as mentioned above) were cultured in EGM-2 supplemented with 20% FBS for 14 d at 37°C with 5% CO_2_. After the culture period (d 14), the EPCs were stained with crystal violet for precise colony demarcation. The number of colonies formed in each set was determined by light microscopy. Colonies were scored on the basis of an accumulation of 15 or more cells. At least triplicate assays were performed for each sample.

### EPC Proliferation Assay

The proliferation of EPCs was determined by 3-(4, 5-dimethylthiazol-2-yl)-2,5- diphenyltetrazolium bromide (MTT) assay. Cells were cultured in 24 well plates (VN-coated and PLLA or PCG-containing) for 14 d and then were incubated with MTT (0.5 mg/ml, Sigma) for 4 h in the dark at 37°C with 5% CO_2_. The purple-blue formazan crystals were dissolved with acidified iso-propanol and the absorbance was measured at 550/650 nm.

### EPC Viability Assay

Viability of the cultured EPCs was assessed using trypan blue dye exclusion test. Data were expressed as % viability.

### EPC Migration Assay

The migratory function of late EPCs in response to a chemotactic cue, a crucial event involved in vasculogenesis, was evaluated by a modified Boyden chamber (BD) assay. In brief, VN-EPCs on d 14 were detached from the wells on d 14 with TPVG (Trypsin, Phosphate, Versene, Glucose, HiMedia, Mumbai, India), and seeded in the upper chamber of 24-well Transwell plates with polycarbonate membrane (8 µm pores) in serum-free endothelial growth medium. PLLA and PCG matrices (along with the cells growing in them) were picked up with sterile forceps and placed ‘face down” into the upper chamber of the inserts. Serum-free DMEM containing VEGF (50 ng/ml) was placed in the lower chamber. After incubation for 24, 48 and 72 h, the membranes were washed briefly with 1×PBS and fixed with chilled 2% buffered paraformaldeyde. The upper side of membrane was wiped gently with a cotton ball. The membrane was then stained using crystal violet for 3 h and then washed with 1×PBS. Cells on the membrane as well as the ones that had migrated into the lower chamber were counted to get the total count of the migrated cells. The magnitude of migration of EPCs was evaluated by counting the total number of migrated cells in each well. At least six replicates were kept in each experiment. Data were expressed as mean % EPC migration ± standard deviation (S.D).

Percent EPC migration was calculated using the following formula:

% migrated EPCs = Total number of migrated cells×100/Total number of cells seeded.

### Induction of Experimental Diabetes and Estimation of Plasma glucose

Mice (6–8 weeks old) were rendered diabetic by administering 120 mg/kg body weight of streptozotocin (STZ; Sigma Chemical Co., St. Louis, USA) via the intra-peritoneal route. STZ was reconstituted in chilled sodium citrate buffer (pH 4.5) just before injection. STZ-treated animals were considered as frankly diabetic after they consistently exhibited random plasma glucose concentrations of more than 200 mg/dL for 2 weeks or more.

### Wound Creation and Closure

Full thickness wounds were created in non-diabetic and diabetic mice. The mice were randomly divided into the following groups: Non diabetic controls; diabetic mice without treatment (diabetic controls); diabetic mice treated with VN-EPCs delivered as a bolus (VN-EPC-treated) and diabetic mice treated with PCG-EPCs (PCG-EPC-treated) or PLLA-EPCs (PLLA-EPC-treated). The methodology was as follows: mice were de-haired and full thickness wounds of 0.75 cm diameter were created on the flanks. Equal number of cells was used for all experimental groups. PCG or PLLA matrix patches (with or without EPCs) of appropriate diameter were applied to wounds with the help of non-medicated, sterile adhesive tapes. Matrix patches were changed every four days. Wounds were washed with antibiotic (Fluconazole, 2 mg/ml, Cipla, Roorkee, India). Morphometric analysis was performed on digital images using the Imaging Software (Q Capture Pro, Olympus, Singapore). The area of wound closure was quantified by processing of photographs taken at various time points and was calculated as the percentage of the wound area immediately after surgery (D0). At least 15 mice were used per group.

### Study of Cellular Profile during Wound Healing

For determination of cellular profile during wound healing, mice were sacrificed on days 2, 4, 6, 8 and 10. Paraffin-embedded wound sections were subjected to Hematoxylin-Eosin (HE) staining for histopathological analysis.

### EPC Incorporation into Wound Bed

EPCs (with or without the PCG/PLLA matrix) were stained with Cell tracker-Orange, a fluorescent probe that freely passes through the cell membrane and is converted to a cell-impermanent fluorescent reaction product in the cytoplasm of live cells. These stained cells were delivered onto diabetic wounds as a bolus for VN-EPCs or by a direct dermal application of dye-loaded PCG−/PLLA-EPCs to the wound beds. Mice were scarified after 48 h and cryo-sectioning was performed on the dissected wound sites. The sections were counterstained with DAPI prior to confocal microscopy analyses. Incorporated cells (CMRL+) were manually counted from a minimum of 15 non-overlapping fields and expressed as percentage of total cells visible in the field (stained with DAPI).

### Data Analyses and Statistical Procedures

Data were expressed as mean ± SD of at least 6 replicates in each experiment. Each experiment was repeated at least thrice. Statistical comparisons were made between groups using one-way ANOVA. The significance of differences between groups was determined by the Tukey post hoc analysis. P value <0.05 was considered as significant. Three independent experiments were analyzed for statistical significance.

## Results

### Structural Characterization of the PCG Matrix

**Figure 1:**
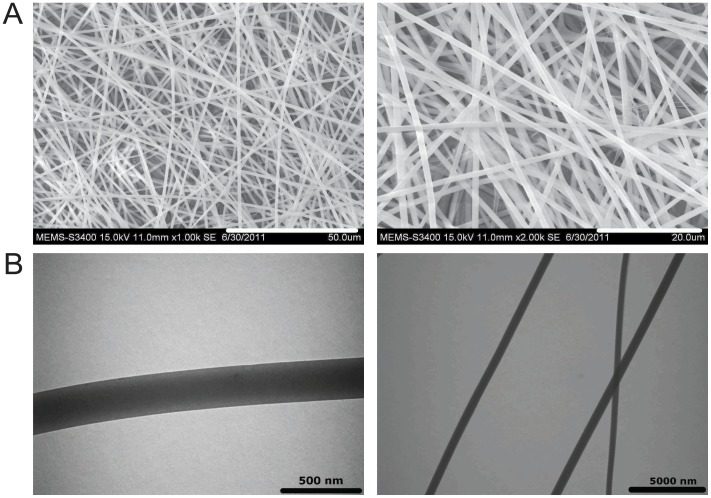
Characterization of PCG-matrix by electron microscopy. (A) SEM images depict morphology of electrospun PCG nanofibers showing random orientation and smooth morphology. Image on the left: 1000× magnification (scale bar = 50 µm) and image on the right: 2000× magnification (scale bar = 20 µm). (B). TEM images of electrospun nanofiber of PCG matrix showing smooth surface (scale bar on left = 500 nm; scale bar on right = 5000 nm).

Post-fabrication, the matrix was subjected to SEM and TEM studies for structural characterization and analysis. The PCG matrix demonstrated a random mesh of uniformly smooth nano- fibers ranging from 400–700 nm in diameters (Figure 1A). TEM examination of the PCG-matrix revealed its smooth morphology, which is important for cell adhesion (Figure 1B). The intricate mesh work of the matrix increases the surface area available for cell attachment and chances of cell entrapment while the smoothness of fibers enhances the chances of cell adhesion. Both these results, thus, highlight the potential applicability of the PCG matrix for cell growth and proliferation.

Having thus ascertained its suitability as a potential substrate for cellular growth, we wished to confirm its structural and functional contribution in propagation and enrichment of functional EPCs from a heterogeneous population of bone marrow-derived MNCs over a 14 d culture period. Few studies, so far, have demonstrated the possibility of direct attachment and enrichment of EPCs from BM-MNCs onto nano-fibrous scaffolds. As a 3D matrix control, we have used a PLLA-based matrix, which has been demonstrated to have similar nanofibrous morphology. The micro-structural characterization of the control scaffold, including SEM and TEM studies, have been published by Jaiswal et al in an earlier study [23].

### The PCG Matrix Promotes Growth and Enrichment of EPCs from the Bone Marrow-derived MNC Population

**Figure 2:**
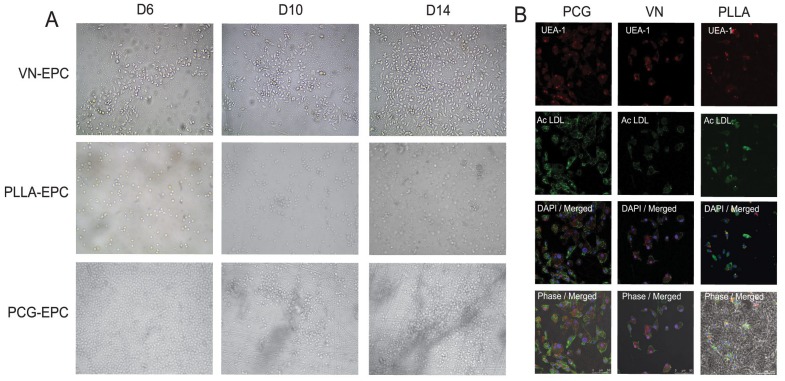
Comparative growth of EPCs on various matrices. (A). A representative phase contrast image of EPCs growing on VN, PLLA and PCG matrix is depicted (200× magnification). (B). Characterization of VN, PLLA and PCG-EPCs. EPCs were co-positive for Alexaflour 488-Ac-LDL uptake and UEA1-TRITC staining. The nuclei are stained with DAPI. Presence or absence of nano-fibre matrix is visible in the phase contrast merged image. (scale bar = 50 µm).

The first step for determination of the efficacy of the PCG-EPCs in terms of cell adhesion and overall growth promotion potential was to compare them with those grown on VN, a standard substrate for EPC culture growth, and with those grown on PLLA matrix control that has been demonstrated earlier to support smooth muscle cell as well as EPC proliferation to some extent [15]. The non-adherent fraction of mouse bone marrow-derived MNCs (1000 cells/well) was seeded onto VN, PLLA and PCG matrix. The heterogeneous cellular population was cultured in EGM2 medium supplemented with 20% FBS for 14 d with replenishment of medium every 48 h. It was observed that all the three substrates supported MNC adherence as well as promoted enrichment of EPC population for 14 d in culture (Figure 2A). Characterization of these cells with Ac-LDL uptake and UEA-1 co-staining confirmed their EPC status (Figure 2B). However, quantitation of these cells revealed that all three substrates had different potential for EPC growth. At d 14, VN showed formation of only 185.75±18.9 EPCs/1000 seeded MNCs, while PLLA yielded a relatively higher number of EPCs (373±45.2 EPCs/1000 seeded MNCs). The PCG matrix proved to be an even better substrate for MNC attachment and EPC formation (529±14.1 EPCs/1000 seeded MNCs), giving approximately 3 times and 1.5 times (p<0.001) higher number of EPCs than VN and PLLA controls, respectively. These findings indicate that the PCG matrix has a higher potential for EPC growth as compared to the controls.

### PCG Matrix does not Affect Stem Cell Status of the Endothelial Progenitor Cells (EPCs)

After having established that the PCG matrix promotes a higher EPC growth compared to the controls, it was imperative to determine whether the substrate had affected the stem cell status of these cells, especially since our initial seeding population was heterogeneous and no pre-selection process was employed. Manual scoring of at least 5 fields from 3 separate experimental sets revealed that 95.68±0.63% of the cells growing on the PCG matrix were dual positive for Ac-LDL uptake and UEA-1 binding, indicating that they had retained their progenitor status. This result was comparable to that found in control cells grown on VN (96.12%). On the other hand, only ∼ 90% of the cells growing on PLLA-matrix were Ac-LDL^+^UEA1^+^ (90.66±4.70%) (p<0.050). The level of EPC enrichment on the PCG-matrix reaches the levels of population enrichment achieved, by other groups, on standard substrates like VN and is a desirable feature in terms of cellular number and population homogeneity [21].

### The PCG Matrix Promotes Cell Adhesion, Colony Formation and Cellular Proliferation along with Maintaining Cellular Viability

A primary concern for such application-oriented systems is the evaluation of the exact efficiency of cell growth and its numerical advantage over the control substrates. In order to make a quantitative assessment of the matrix potential, we further tested the efficacy of the PCG matrix by comparing the three groups using a panel of standard in vitro assays comprising of cell adhesion assay (CAA), colony formation unit assay (CFU), proliferation potential assay (MTT) and % viability using the trypan blue dye exclusion test.

As mentioned in the materials and methods section, MNCs were seeded onto control and test matrices and the results were analysed on d 14.

**Figure 3:**
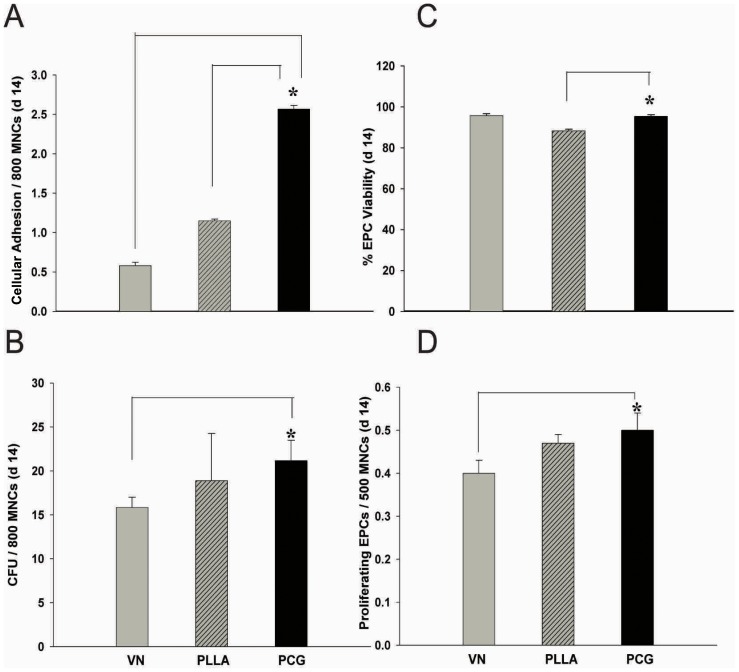
Biocompatibility assessment of PCG- matrix Biocompatibility of the PCG matrix was assessed by estimation of cell adhesion (A), colony forming cells/units (B), % viability (C) and proliferation potential (D) on d 14, in comparison with the parallel VN- and PLLA-grown EPCs used as controls. Data are represented as mean of triplicate wells from 5 independent experiments (n = 5) ± SD. *p<0.001.

While all the three substrates allowed MNC adhesion and EPC formation (Figure 3A), the electro-spun matrices, being 3D systems, demonstrated a higher cellular adherence compared to VN – a 2D system (PLLA: 2.16- times and PCG: 4.42 times higher) (p<0.001). Most interestingly, however, the PCG matrix showed a better capacity for cellular adhesion compared to the matrix control, PLLA (2.04 fold higher than PLLA; p<0.001). These findings point towards the enhanced ability of the PCG matrix for cellular entrapment and/or attachment. This is an interesting observation and highlights the applicability of the PCG matrix for achieving high numbers of EPCs per unit area. It is possible that the components, polycaprolactone and gelatin, in the optimized ratio (3∶1) may be responsible for the high cellular adherence exhibited by the PCG-matrix. The larger surface area provided by the PCG matrix, its specific composition as well as the entrapment of the seeded cells in the nano-fibre mesh together may have contributed to the higher cellular adherence and growth obtained with it.

The CFU assay, too, revealed a similar story. Both the electrospun matrices, PCG and PLLA, demonstrated higher colony formation as compared to VN (1.33 and 1.10 fold respectively) (Figure 3B; p<0.001). The average colony size contributes to effective population size and hence is an important parameter in the CFU assay. We found that the average colony size on the PCG matrix (24.16±2.56 cells per colony) was larger than that on VN (17.66±1.63 cells/colony) as well as PLLA controls (20.27±2.74) (p<0.010). This is an important observation as a combined effect of active cellular adherence to the matrix fibres and enhanced cell growth, indirectly leading to a larger colony, effectively translates into a higher cell number, which is one of the most desirable attributes necessary in the development of such application-oriented scaffold systems.

Corroborating the earlier observations, the MTT assay revealed that both the nano-fibrous matrices, PCG and PLLA, promote higher EPC proliferation compared to VN (1.25 and 1.17 fold respectively). These data indicate that the PCG matrix not only actively enhances adherence and colony formation, but also augments the proliferation potential of the attached cells (Figure 3D).

All these promising results would be rendered inconsequential if the overall viability of EPCs was compromised/adversely affected due to long term culture on the test matrix. Encouragingly, viability assessment revealed that both VN-EPCs and PCG-EPCs showed a high % viability, while the PLLA matrix control demonstrated a significantly lower % viability, (Figure 3C; p<0.050). The data demonstrate that PCG matrix retains the EPC viability at par with vitronectin. This feature is important for long term propagation of cells for applications such as delivery onto wound or injury sites.

### Active Interaction of EPCs with the PCG Matrix

**Figure 4:**
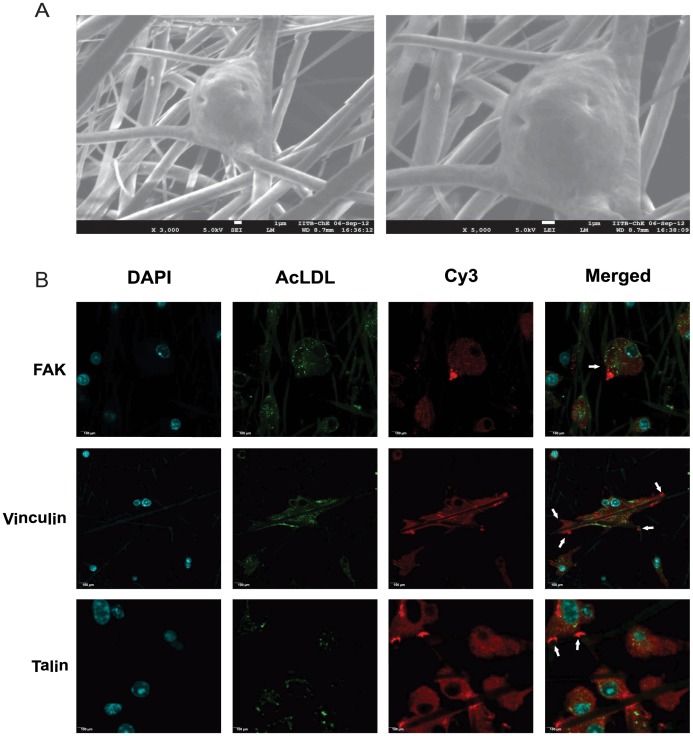
Formation of focal adhesions by EPCs on PCG-matrix. (A) SEM images of PCG matrix-embedded EPCs adhering to matrix fibres by cellular processes. Magnification of images: Left: 2,200 X; Right: 5,000 X. (B) Confocal analyses of PGC-EPCs dual stained for Ac-LDL-Alexaflour 488 and Focal Adhesion Kinase (FAK), Vinculin and Talin. All secondary staining was done with Cy3. Nucleus is stained with DAPI. Scale bars for images = 100 µm.

Since adherence of cells onto the matrix plays a crucial role in attaining high cell numbers, we determined whether the enhanced adherence of EPCs on the PCG-matrix was mediated through active cellular mechanisms or was a consequence of passive cellular entrapment in the nanofiber mesh. SEM analyses of the PCG matrix (along with the EPCs) revealed that the cells actively held onto the matrix fibres with the aid of “cellular processes/extensions” (Figure 4A). To shed further light on the phenomenon, we stained the PCG-EPCs with antibodies to Focal Adhesion Kinase (FAK), Vinculin and Talin – all of which are known to accumulate at the focal adhesions. Confocal microscopy analyses of the same revealed that FAK, Vinculin and Talin specifically localized, and were strongly expressed, at the sites where the cells were actively holding onto the matrix fibres i.e. forming active focal adhesions (Figure 4B; white arrows indicate focal adhesion sites). Formation of such focal adhesions was not seen in VN-EPCs or PLLA-EPCs (Figure S1). These results show that the enhanced adherence observed in relation to the PCG matrix is attributable to active focal adhesions formed by the EPCs at the sites of matrix interaction – an added plus for its biocompatibility quotient.

### The PCG Matrix Allows Sustained in vitro Migration of EPCs

The active interaction of EPCs with the PCG-matrix reduces the chances of cellular loss via flow-away mechanisms when applied onto delivery sites, but it simultaneously raises a possibility that it may not allow chemotactic migration of EPCs. Such a situation would result in a substantial loss of its therapeutic applicability.

**Figure 5:**
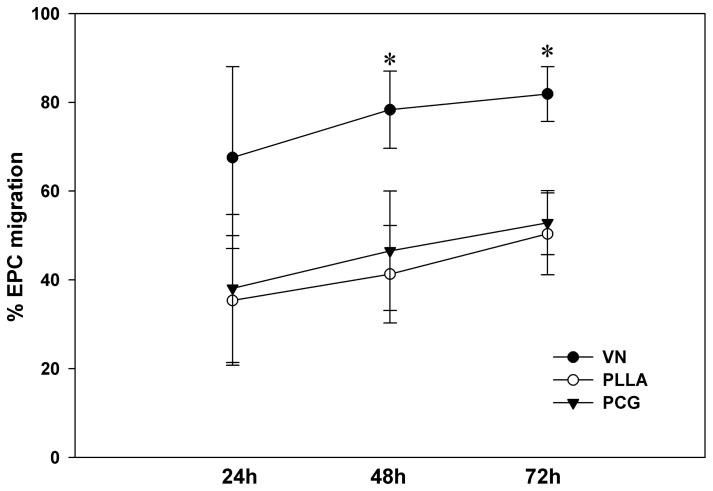
Chemotactic migration of EPCs grown on various matrices. Comparative EPC migration towards VEGF (50 ng/ml) in vitro at 24, 48 and 72 h from VN, PLLA and PCG matrix is graphically illustrated. Data are represented as mean of at least 3 independent experiments (n = 3) ± SD. Each experiment had at least 4 wells per group. EPCs from both electro-spun matrices consistently exhibited a slower cellular migration compared to those from VN at all time points (*p<0.001).

To examine this issue, we set up a migration assay at various time points. It was observed that both the electro-spun matrices (PCG and PLLA) allowed slow, but sustained chemotactic migration of cells. A total of 52.87±7.24% of PLLA-EPCs and 50.36±9.2% of PCG-EPCs migrated by 72 h while 81.87±6.17% of VN EPCs had migrated by this time (Figure 5; p<0.001). These data show that ∼ 50% of the seeded EPCs were retained *in situ* till 72 h by the electrospun matrices, suggesting that that these matrices, due to their mesh-like structure, act as physical barriers to cell movement, thereby retarding, but not preventing, the chemotactic migration of EPCs.

The aforementioned results raise a question about possible reduction in viability of the EPCs as a consequence of the migration-induced shear stress. So we monitored the viability of migrated EPCs using trypan blue dye exclusion test. We found that the migrated VN-EPCs showed a significant reduction in % viability over the 24, 48 and 72 h of migration (92.6, 86.24 and 76.58% respectively), while PCG (95.3, 90.37 and 87.45% respectively) and PLLA (93.11, 90.51, 84.84% respectively) prevented the reduction in viability under similar conditions (Table 1; p<0.001). Although this finding deserves additional experiments to determine the mechanistic aspects, we hypothesize that the electro-spun matrix-entrapped cellular secretory products may cushion the EPCs against migratory stress-induced cell death. Perhaps, the flexibility of the electrospun nano-fibers may also contribute to the reduction in shear stress.

**Table 1:** % viability of migrated cells at 24, 48 and 72h.

Time Point	VN-EPCs	PLLA-EPCs	PCG EPCs
24 h	92.6±20.0	93.11±12.8	95.36±12.5*
48 h	86.24±16.5	90.51±13.6	90.37±51.2*
72 h	76.58±32.6	84.84±18.8	87.45±37.2*

PCG-EPCs exhibited higher viability post-migration as compared to VN and PLLA-EPCs. Although a progressive decrease in % cellular viability was observed over 24, 48 and 72 h time points in all groups, PCG-EPCs show significantly higher maintenance of cellular viability as compared to its PLLA and VN-EPC counterparts at all time points (*p<0.001). Results are represented as mean ± SD of at least 3 independent experiments (n = 4/5).

Since the final aim of the experiment is the delivery of live EPCs onto diabetic wounds, it was important to assess the correct number of live deliverable EPCs obtained with the three substrates used. After pre and post migration correction, the effective number of live deliverable EPCs/1000 MNCs seeded was: 135 for VN; 279 for PLLA and 442 for PCG (p<0.001) i.e. the effective dose of live deliverable cells in the PCG-matrix was 1.58 times higher than PLLA and 3.27 fold higher than VN (p<0.050; Table S1).These data show that the PCG matrix scores the highest in this aspect, thus underscoring its potential for clinical application in wound healing studies as well as other studies requiring a controlled and/or sustained delivery of live cells.

### Topical Application of Matrix-embedded EPCs onto Wounds in Diabetic Mice Enhances Wound Healing

**Figure 6:**
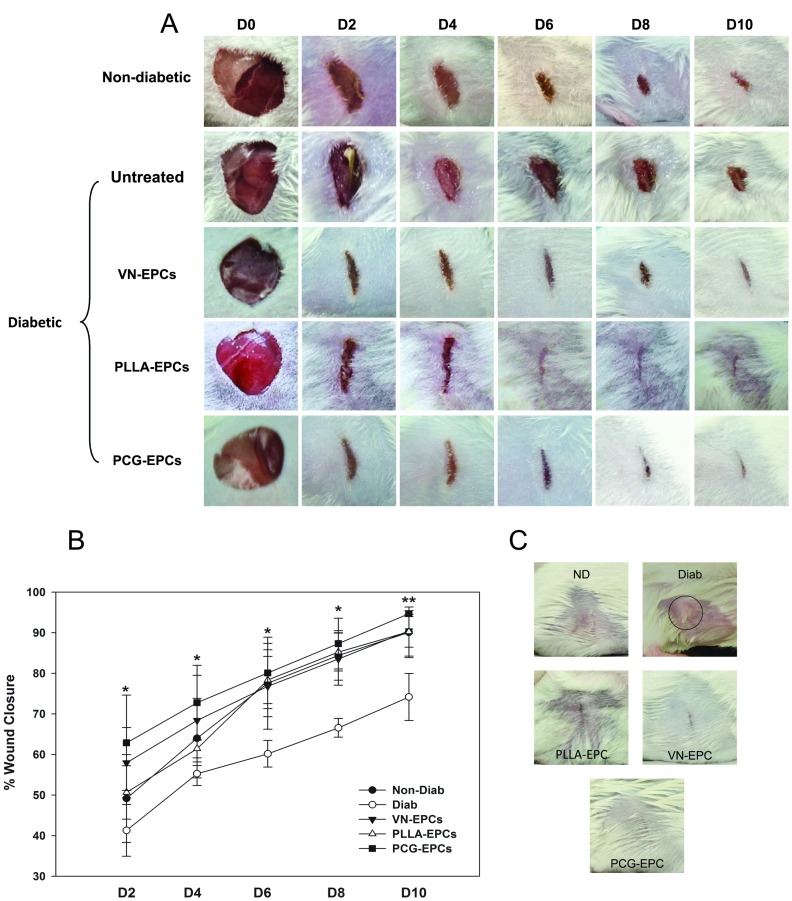
(A) PCG-EPCs accelerate wound healing process. Photographic evidence of wound healing in non-diabetic animals in comparison to untreated diabetic, VN-EPC-treated, PLLA-EPC-treated and, PCG-EPC-treated diabetic animals, from d 0 to d 10. Photographs are from one representative experimental set from among 3. At least 3 mice were analyzed for each time point. (B). Graphical representation of percent wound closure in various groups. Percent wound closure in PCG-EPCs is significantly higher compared to VN-EPC-treated, PLLA-EPC-treated and control groups on days 2 to 8 (*p<0.010) as well as on day 10 (**p<0.050). (C) Photographic evidence of scar tissue formation in non-diabetic (ND), untreated diabetic (Diab), VN-EPC-treated (VN-EPC), PLLA-EPC-treated and PCG-EPC-treated diabetic mice (PCG-EPCs) on day 22. Fibrotic tissue formed in untreated diabetic mouse is encircled in black. The PCG-EPC-treated wounds healed without the formation of any scar tissue or deposition of collagen. The data are representative of at least 4 mice per group.

Our in vitro data clearly demonstrate that the PCG matrix is an effective tool for enhanced EPC growth and delivery. We further determined whether the potential evident in the in vitro studies translates into in vivo applicability in diabetic wounds by performing standard in vivo wound healing assays in mice. For this purpose, full thickness wounds were created in untreated diabetic controls as well as test diabetic animals and the wound healing process was studied by measuring the wound closure dynamics and performing histopathological analyses on days 2, 4, 6, 8 and 10. Non-diabetic animals that were wounded and sacrificed at the same time points were used as non-diabetic controls. As expected, the untreated-diabetic-control animals (Figure 6A; second panel from top) exhibited a delayed wound healing as compared to the non-diabetic controls (Figure 6A, uppermost panel). Application of the VN-EPCs (Figure 6A; Diabetic- VN-EPCs –third panel from top), PLLA-EPCs (Figure 6A; Diabetic- PLLA-EPCs –fourth from top) as well as PCG-EPCs (Figure 6A; Diabetic-PCG-EPCs; lowermost panel) to the diabetic wound site improved the rate of wound healing and a near-complete wound closure was observed by d 10.

The percentage wound closure graph revealed that the VN-EPC- and PCG-EPC-treated diabetic groups showed a significant improvement in wound healing from d 2 itself (57.96±8.6 and 62.88±11.7 respectively; Figure 6B), as opposed to the non-diabetic and the diabetic control groups, underscoring the salutary effect of EPCs in the wound healing process. However, it must be noted here that PLLA-EPC-treated diabetic animals showed a delayed would healing on d 2 (50.65±6.58) and d 4 (61.43±2.25). By day 10, the non-diabetic controls, the VN-EPC-treated and PLLA-EPCs showed a comparable % wound closure (90.1±6.24, 90.47±4.06 and 90.26±6.04 respectively, differences not significant) while the PCG-EPC-treated experimental mice showed a consistently higher percent wound closure at all time points, ultimately achieving a significantly higher percent wound closure (94.77±2.6%; p<0.050) compared to both VN- and PLLA-EPCs. The diabetic control, as expected, did not achieve complete wound closure by day 10 (74.16±5.8%).

### Application of PCG-EPC Leads to Fibrosis-free Wound Healing

In addition to the wound closure dynamics, it is also important to determine the quality of the healed wounds. An important indicative of a poor quality wound healing is the formation of fibrotic scar tissue post wound closure. To study this parameter we permitted the created wounds from all groups to heal completely and allowed an additional follow up period of 10 days post wound closure for scar tissue to form. All animals were then photographed for fibrosis/scar formation on day 22 (Figure 6C). An intense fibrotic tissue scar formation (encircled) was seen in the healed diabetic wounds that did not receive EPCs of any kind. On the other hand, the diabetic animals treated with VN-EPCs showed only a marginal scar formation. The diabetic mice treated with PLLA-EPCs did not show scar formation, but showed an irregular skin healing, possibly due to local collagen accumulation. On the other hand, the diabetic mice treated with PCG-EPC showed no scar tissue formation or collagen deposition and exhibited a complete, clean and uniform wound healing that was comparable to the non-diabetic controls. Taken together, these in vivo wound healing data clearly revealed that the application of PCG-EPCs on the diabetic wound site not only enhances the rate of wound healing, but also improves the quality thereof.

### PCG-EPC-mediated Wound-healing Simulates Normal Wound Healing Process

**Figure 7:**
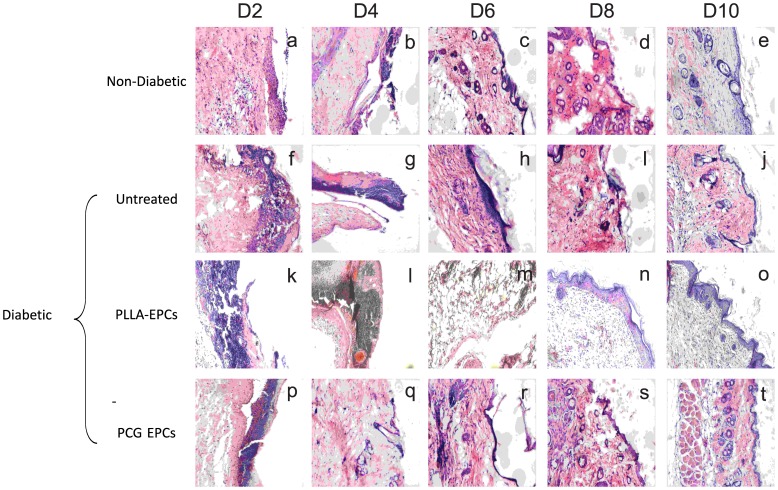
PCG-EPC-treated wound healing mimics normal wound healing process. Histopathological analyses of wound healing process from d 2 to 10. Non-diabetic controls (Figure 7; images ‘a’ to ‘e’), untreated diabetic controls (Figure 7; images ‘f’ to ‘j’), PLLA-EPC-treated diabetic wounds (Figure 7; images ‘k’ to ‘o’) and PCG-EPC-treated diabetic wound (Figure 7; images ‘p’ to ‘t’) (10× magnification) show that PCG-EPC-treated wounds mimic normal wound healing process. All data are representative of at least 3 independent experiments with at least 3 mice per time point.

We have demonstrated so far that the PCG matrix is not only a better substrate for growth and enrichment of EPCs, but is also an effective delivery system for the topical application on wounds for accelerated and scar-free healing. However, it was important to examine whether the cellular profile of the wounds treated with PCG-EPCs was comparable with the non-diabetic wound healing process. This would indeed be the ultimate test of the efficacy of the system. For this purpose, we performed sequential histopathological analyses of the untreated diabetic control and PCG−/PLLA-EPC-treated diabetic wounds and compared the same with the wound healing profile of the non-diabetic untreated wounds – representing the normal wound healing process (Figure 7). The time points chosen for this study were days 2,4,6,8 and 10. At d 2, all wounds showed localized inflammation and macrophage infiltration, a scenario expected in a relatively fresh wound. As expected, this was particularly intense in the diabetic wound sections (Figure 7; image ‘f’). Wounds in the PLLA-EPC-treated mice demonstrated an intense inflammation on day 2 (Figure 7; images ‘k), while non-diabetic and PCG-EPC treated groups showed only mild inflammation (Figure 7; images ‘a’ and ‘p’).

It is known that granulation is the first step towards wound healing. Sections of non-diabetic controls as well as PCG-EPC-treated diabetic wounds on day 4 showed a reduction in inflammation and mild granulation (Figure 7; images ‘b’ and ‘q’ respectively). These events were not seen in the untreated diabetic sections and, surprisingly, also in the PLLA-treated diabetic sections, as the inflammation had not completely diminished by day 4 (Figure 7; image ‘g’ and ‘l’). The inflammation seen on days 2 and 4 and the delayed onset of granulation seen in these mice corroborated with the delay in percent wound closure observed in them. By d 6, the non-diabetic wounds had recovered sufficiently well to develop a few secondary structures and a comprehensive epithelial lining (Figure 7; image ‘c’). The PLLA-EPC-treated wounds demonstrated some degree of recovery and granulation by this time point (Figure 7; image ‘m’). The PCG-EPC-treated wounds showed the level of recovery that was comparable to that seen in the non-diabetic animals and the re-epithelialization process was already apparent (Figure 7; image ‘r’). The diabetic untreated wounds, on the other hand, still showed signs of mild inflammation and infiltration. However, mild epithelialization and granulation were also visible in these sections (Figure 7; image ‘h’). On d 8, comparably progressive wound healing was observed in the non-diabetic and the PCG-EPC- treated diabetic wounds, including rapid epithelialization and secondary structure formation (Figure 7; images‘d’ and ‘s’ respectively). The PLLA-treated sections showed improvement in the wound condition, including formation of a few secondary structures along with the formation of a thick epithelial lining (Figure 7; image ‘n’). The diabetic wounds by day 8 began to show traces of granulation and epithelialization (Figure 7; image ‘i’). By day 10, the untreated diabetic wounds demonstrated some degree of epithelialization, but very little secondary structure formation was observed and the quality of wound healing was poor (Figure 7; image ‘j’). By d 10, the wounds in non-diabetic animals and PCG-EPC treated diabetic animals had completely healed (Figure 7; images ‘e’ and‘t’). However, in the PLLA-EPC-treated diabetic wounds, mild granulation as well as thickened epithelial lining was still evident, indicating a delayed wound healing (Figure 7; image ‘o’).

It is thus clear that the cellular profile of the PCG-EPC-treated diabetic wounds faithfully mimics the healing profile of the non-diabetic wounds. These data indicate that though the difference in % wound closure between the PCG-EPC-treated and control sets appears small (but significant), the sequential histo-pathological analyses clearly show that the PCG-EPCs have a major impact on wound healing.

### PCG-EPCs Show Efficient Incorporation into the Diabetic Wounds

**Figure 8:**
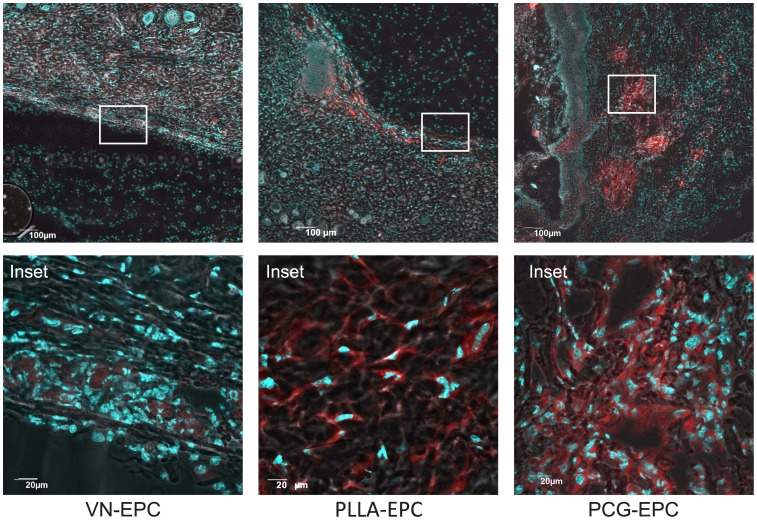
PCG-EPCs migrate deep into the wound beds. VN-EPCs (Figure 8; images ‘a’ and ‘d’), PLLA-EPCs (Figure 8; images ‘b’ and ‘e’) and PCG-EPCs (Figure 8; images ‘c’ and ‘f’), pre-stained with cell tracker-Orange, were delivered at d 0 onto diabetic wounds for 48 h. Cryosections were counterstained with DAPI and analyzed for incorporation of pre-stained EPCs into wound beds of diabetic mice. Scale bars for images = 100 µm; Scale bars for insets = 20 µm. The data are representative of 4 mice in each group.

From the data obtained so far it was clear that application of the PCG- EPCs results in accelerated and scar-free healing of diabetic wounds. We speculated that this could be due to a better incorporation of the PCG-EPCs into the wound beds compared to the VN-EPCs delivered en-bolus. In order to examine this possibility, we loaded the EPCs with a vital fluorescent dye, Cell Tracker-Orange, and then applied them onto the diabetic wound beds (0 h) as was done in the previous experiments. These mice were sacrificed at 48 h, since maximal EPC incorporation into the wounds was expected to occur by this time point (Figure 6B). Wound beds from all mice were cryosectioned and stained with DAPI to demarcate the nuclei. Since the original wound tissue was not pre-stained with the CMRL Orange dye, it is evident that the stained cells would have migrated from the matrices into the wound bed. It was observed that the number of CMRL^+^ cells (red coloured) in the sections obtained from the PCG-EPC- or PLLA-EPC-treated wounds was significantly higher (84.48±3.78%/field and 77.76±7.62% cells/field respectively, Figure 8) compared to those in the sections obtained from VN-EPC-treated wounds (59.80±4.50%/field). Merged phase contrast images revealed that the PCG- and PLLA-EPCs were not merely assorted cells lying on top of the wound bed, but were actively incorporated in the wound tissue (Figure 8; PCG-EPC inset, PLLA-EPC inset). The PCG-EPCs were found to have migrated deep into the wound bed while the PLLA-EPCs were localised closer to the surface (Figure 8; images ‘c’ and ‘b’ respectively). On the other hand, the VN-EPCs were only superficially present at the wound edge and had not migrated deep into the wound-tissue (Figure 8; VN-EPC inset). This may be a combined effect of bolus wash-off as well as post-migration decrease in viability.

## Discussion

One of the serious complications seen in DM is the impairment of EPC-migration towards wound sites. It was discovered that the delivery of non-diabetic EPCs effectively contributes to wound rescue in experimental diabetic models. Published data suggest that larger the number of EPCs transferred onto the wounds, either by natural processes or when applied externally, faster is the process of wound healing [4]–[9]. However, all the externally applied/delivered cells do not necessarily get incorporated into healing wound patches, primarily because of the “wash away” mechanisms. To rectify these lacunae and to amplify EPC incorporation, efforts have been directed towards optimization of EPC culture and cellular transfer to wounds using a variety of delivery systems. Some of the latest efforts include delivery of EPCs onto wounds using various bio-artificial scaffolds, seeded with pre-cultured EPCs prior to delivery. Such scaffolds have been used successfully for a wide range of cell and model types, including diabetic wound healing models [24]–[33]. However, very few studies demonstrate the efficacy of utilizing an electrospun nano-fibrous scaffold in effective healing of diabetic wounds.

One of the major drawbacks associated with most of these matrices is the requirement for seeding pre-isolated/cultured EPCs, and subsequent adherence onto the matrix, prior to delivery onto the wound sites [34]–[36]. In any clinical setting, an increase in the handling steps not only increases the chances of contamination, but also adds to the production cost. Secondly, although many matrices promote the adhesion of the EPCs, they may/may not function as an effective delivery system for various reasons, thereby reducing their direct in vivo applicability [37], [38]. To overcome these lacunae, we fabricated a relatively simple, stable, strong and inexpensive electrospun biocompatible matrix using an optimized combination of polycaprolactone and gelatin, which comprehensively performs both these functions. Electro-spinning enables the making of nanofibrous matrices having high surface-area to volume ratio along with high flexibility, strength and porosity. It is understood that such matrices are suitable for wound healing applications as they can be comfortably draped over the wounds, allow permeation of atmospheric oxygen to the wound site and enable draining of the wound exudates [39]. Polycaprolactone, in combination with other bio-compatible substances, has been used for tissue engineering applications with promising results previously [40]–[43]. Here, we have optimized a novel ratio of Polycaprolactone: Gelatin (at 3∶1) for the fabrication of an electrospun mesh that not only supports cell growth, but also allows the removal of dead cells and cellular exudates from the spent medium. Since removal of exudates is a major concern in long term 3D cultures, this would be an important attribute in the current 14 d EPC culture and enrichment system. This may also have an added advantage for diabetic wound healing, as these wounds are known to be hypoxic, and need to be plentifully supplied with oxygen and must allow the removal of exudates for better wound healing. In this context the aforementioned properties may indeed be beneficial for chronic wound healing in general and diabetic wound healing in particular.

Most substrates allow attachment or at the most short-term maintenance of the pre-isolated/enriched EPCs post-seeding [44]. The CFC assay (Figure 3B), which reflects lineage specificity, indicates that the PCG matrix actively promotes significantly higher EPC growth under “media-induced selective conditions” compared to VN and PLLA. This is a unique feature of PCG matrix, as most known matrices require seeding of pre-isolated EPCs while the PCG matrix allows their enrichment and proliferation over a 14 d period- post seeding of a heterogeneous population. To the best of our knowledge, the PCG matrix is the only matrix that allows direct seeding, enrichment and expansion of EPCs in culture, and hence qualifies as a candidate for further research as a growth substrate for EPCs. As demonstrated by SEM analysis (Figure 4A), the PCG matrix actively promotes a higher cellular attachment and colony formation. Confocal microscopy analyses further revealed that the EPCs actively hold onto the PCG matrix by the formation of focal adhesions. This active adhesion is perhaps an important contributory factor in the high levels of EPC attachment observed in the case of PCG-matrix. This could be due to the incorporation of optimal concentration of gelatin, a denatured form of collagen, which is known to enhance cell to cell and/or cell to substrate adhesion [45]. Similarly, passive entrapment of EPCs in the matrix pores may also play a role in the high cell numbers. A Z-stack image analysis of the PCG-matrix-grown EPCs revealed DAPI-stained cells entrapped at all levels throughout both the matrices (data not shown). Since active adhesion, along with entrapment, prevents wear and tear-induced cell loss, the effective cell number retained in the matrices may be considerably higher than those cultured on VN. It is evident from these findings that the electrospun matrices promote attachment as well as entrapment of cells, resulting in a significantly higher cell number per unit surface area.

We find a formation of larger colonies with higher level of cellularity in the PCG matrix as compared to the controls. Simultaneously, we also find a higher proliferation of EPCs on the PCG-.matrix. Although we do not rule out the possibility, it is debatable whether these results can be attributed to gelatin alone. It is possible that the autocrine effects of the EPC secretome may have played a role in these enhanced effects. A similar phenomenon has been previously demonstrated by Jokinen et al. [46]. This is an interesting possibility and we are actively delving into the intricacies of the same.

The possibility of population heterogeneity would reduce the advantage of matrix usage for cell growth and enrichment. However, confocal microscopy analyses confirmed that the PCG-matrix-grown cells consisted primarily of ‘true’ EPCs. The ‘enrichment’ from the original seeding population of MNCs may be attributed to the use of EGM2, an EPC-specific positive selection medium, and the matrix itself may not have actively contributed to the selection of cells, though we at present do not completely rule out such a possibility.

Any matrix that allows a firmer attachment of EPCs, may also interfere with the mobility of the cells, thus reducing its in vivo applicability. However, our in vitro migration assay demonstrated that the PCG matrix allows a slower, but a sustained migration towards the chemotactic cues without affecting the viability, indicating its usefulness in application on diabetic wounds. The same was reflected in the in vivo studies wherein a large number of PCG-EPCs were found to have migrated deep into the wound bed. It is possible that the focal adhesion-mediated signaling has imparted a more active phenotype on them. This possibility needs to be formally examined.

The PCG-matrix, thus, not only allows enrichment and growth of EPCs directly from the bone marrow-derived heterogeneous MNC population, but also allows a sustained chemotactic migration of these cells without affecting their viability. This capacity is decidedly an advantage as it simplifies the procedure of ‘bandage creation’, reducing the multitude of steps including isolation, culturing, post-culture retrieval from the growth substrate and transfer onto the delivery system; all of which contribute to the escalation of the cost and may also compromise sterility.

The PCG matrix allowed the placement of the matrix-grown EPCs in an ‘as-is’ manner onto the diabetic wounds. This is an important achievement as most commonly used scaffolds do not allow such ease of growth and applicability. Once ectopically applied like a bandage, the matrix allowed migration of EPCs onto the wound site. Our data (Figure 6B and Figure 8) demonstrate that the PCG -EPCs migrated maximally and got incorporated into the diabetic wound bed during the first 48 h – the most crucial time when maximal numbers of EPCs are recruited towards the wound site – and consequently bring about wound closure comparable to that of the non-diabetic controls. Although similar number of VN-EPCs was delivered as a bolus, fewer cells were found to be incorporated in the wound bed at 48 h (Figure 8). This would certainly be due to the ‘wash off’ events typically associated with bolus delivery. On the other hand, both the PCG, and PLLA matrices allowed a slow, but a sustained delivery of EPCs on days 4, 6, 8 and 10 resulting in an improved wound healing of diabetic wounds. Our data also demonstrate that the PCG-EPC-treated wound healing mimics the normal wound healing pattern (Figure 7) [7]. These findings are further strengthened by our observations pertaining to enhanced incorporation of PCG-EPCs (Figure 8).

It is well established that once the extrinsically delivered EPCs get incorporated into the wound sites, they accelerate the wound healing process [47]–[50]. Beyond all doubts, the PCG-EPCs enhanced the quality of wound healing, as compared to the untreated diabetic wounds, as shown by formation of secondary structures. Additionally, the PCG-EPC treatment lead to a hypertrophic-scar-free, fibrosis-free healing of the diabetic wounds (Figure 6C).

On the other hand, the PLLA matrix supported the EPC growth abundantly, but its in vivo success was found to be limited. Although the PLLA-EPCs got incorporated into the healing wounds during the first 48 h, similar to the PCG-EPCs (Figure 8, PLLA-EPCs and inset), the histopathological time point studies show that there is an intense inflammation on d 2, which does not subside by d 4 as expected (Figure 7, images ‘k’ to ‘o’). These findings are further corroborated by the graphical evidence showing a delay in wound healing at the first two time points (d 2 and 4), after which the delivered EPCs contributed towards wound healing (Figure 6B). It is possible that the initial inflammation may have affected the quality of the healed wound (Figure 7; images ‘k’ and ‘l’ and Figure 6C respectively). It must also be noted here that the PLLA matrix sticks to the wounds and often causes trauma while changing of matrix-dressing (data not shown). It is possible that the initial inflammatory response, along with the dressing-associated-trauma may have resulted in irregular wound healing observed in this set (Figure 6C). On the other hand, the PCG-EPCs did not elicit any inflammatory response (Figure 7, images ‘p’ to‘t’) and also showed consistent, clean and scar- free wound healing (Figure 6C). It is possible that the migration of EPCs deep into the wound bed at an early stage, as seen in the case of PCG-EPCs, may be necessary to achieve a near-normal healing of the diabetic wounds. The PCG-matrix does not stick to the wound and hence changing of dressing is a trauma-free procedure. These factors certainly enhance its therapeutic applicability.

To the best of our knowledge, the PCG-EPC is the only system that allows positive selection and proliferation of EPCs directly from the seeded MNCs. In addition, it is the only scaffold-cell system that allows a sustained delivery of cells onto diabetic wounds during the first 48 h, the crucial time frame, leading to an enhanced and pathologically significant wound healing.

Healing of wounds in a normal healthy animal usually proceeds uneventfully and the problem is showing that the treatment is actually significantly better to warrant the expense and trouble of the treatment. However, in a disease state, such as diabetes, where healing is not normal, the development of an expensive treatment is certainly justified. In this study we have shown that PCG-EPCs bring about an accelerated fibrosis-free wound healing that is demonstrably better than the treatment controls. With these encouraging data in hand we propose to perform these studies on human samples using immune-deficient mice with an ultimate aim of developing a clinical protocol for the treatment of dermal wounds in human subjects.

### Conclusions

Healing in diabetic wounds is difficult and its management is crucial, as an unhealed wound can cause intense pain and trauma. The PCG-matrix facilitates the growth and enrichment of EPCs upon direct seeding of MNCs leading to an accelerated and fibrosis-free healing of diabetic wounds. We have successfully created a ‘ready-to-use EPC growth and delivery system’ that combines the ‘bio-compatibility’ required for growth and enrichment of EPCs with ‘ease of application’ necessary for therapeutic applications. This matrix, thus, has good applicability in the management of diabetic wounds. Our data suggest that similar benefits may also be, potentially, observed in other wounds such as burns, other non-diabetic wounds as well as cosmetic surgical applications.

## Supporting Information

Figure S1 **EPCs do not form focal adhesions on vitronectin or PLLA.** Confocal analyses of VN-EPCs (A) and PLLA-EPCs (B) dual stained for Ac-LDL-Alexaflour 488 and Focal Adhesion Kinase (FAK), Vinculin and Talin. All secondary staining was done with Cy3. Nucleus is stained with DAPI. Scale bars for images = 100 µm
(TIF)

Table S1 **Determination of total number of live, deliverable cells from 1000 mMNCs (after correcting for pre and post migration reduction in viability) from VN, PLLA and PCG matrices.**
(DOCX)
