# Supplementary figures and images for: Enhanced Growth of Endothelial Precursor Cells on PCG-Matrix Facilitates Accelerated, Fibrosis-Free, Wound Healing: A Diabetic Mouse Model
Source: PLoS One. 2013 Jul 26;8(7):e69960. doi: 10.1371/journal.pone.0069960 (PMC3724903; doi:10.1371/journal.pone.0069960)

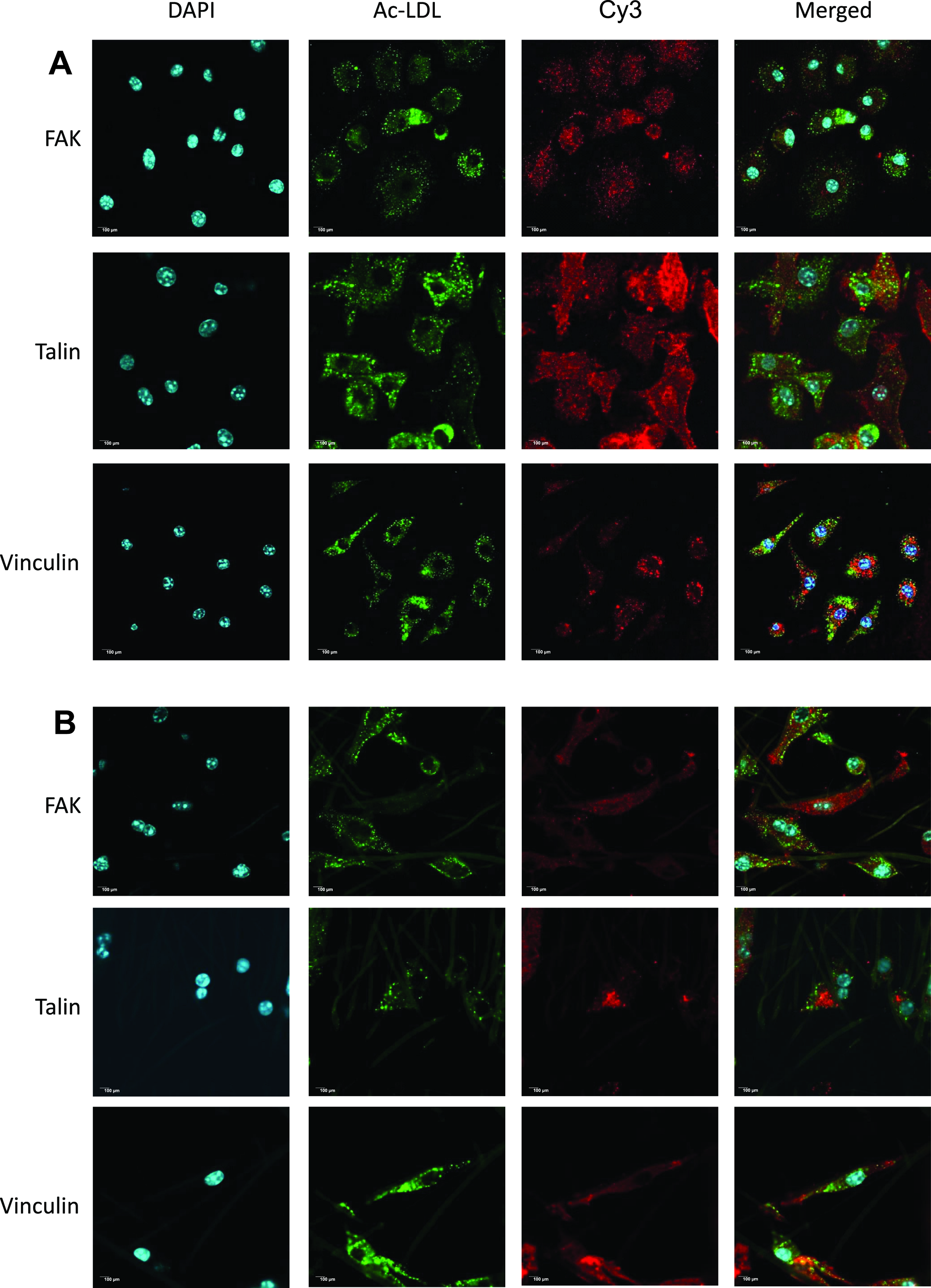

Supplement: Figure S1 — EPCs do not form focal adhesions on vitronectin or PLLA. Confocal analyses of VN-EPCs (A) and PLLA-EPCs (B) dual stained for Ac-LDL-Alexaflour 488 and Focal Adhesion Kinase (FAK), Vinculin and Talin. All secondary staining was done with Cy3. Nucleus is stained with DAPI. Scale bars for images = 100 µm (TIF) [file pone.0069960.s001.tif]
