# Supplementary material for: Enhanced Growth of Endothelial Precursor Cells on PCG-Matrix Facilitates Accelerated, Fibrosis-Free, Wound Healing: A Diabetic Mouse Model
Source: PLoS One. 2013 Jul 26;8(7):e69960. doi: 10.1371/journal.pone.0069960 (PMC3724903; doi:10.1371/journal.pone.0069960)
Supplement: Table S1 — Determination of total number of live, deliverable cells from 1000 mMNCs (after correcting for pre and post migration reduction in viability) from VN, PLLA and PCG matrices. (DOCX) [file pone.0069960.s002.docx]

**Supplementary information.**

**Table S1.**

|  | VN | PLLA | PCG |
| --- | --- | --- | --- |
| EPCs obtained from 1000 seeded MNCs (d14) | 185 ± 18.9 | 373 ± 45.2 | 529 ± 14.1 |
| Live EPCs / 1000 seeded MNCs (d14) | 166 ± 13.6 | 329 ± 15.8 | 504 ± 16.8 |
| Total migrated EPCs | 95.44 ± 13.6 | 173 ± 3.1 | 253 ± 6.9 |
| Total viable migrated EPCs | 72 ± 14.5 | 139.73 ± 21.5 | 221 ± 12.3* |
| Total viable EPCs expected to enter the wound bed/1000 MNCs seeded | 135 | 279.43 | 442 |
